# Comparative Genomic Analysis of a *Methylorubrum rhodesianum* MB200 Isolated from Biogas Digesters Provided New Insights into the Carbon Metabolism of Methylotrophic Bacteria

**DOI:** 10.3390/ijms24087521

**Published:** 2023-04-19

**Authors:** Xi Zhang, Liqing Xia, Jianyi Liu, Zihao Wang, Yanni Yang, Yiting Wu, Qingshan Yang, Luodong Huang, Peihong Shen

**Affiliations:** State Key Laboratory for Conservation and Utilization of Subtropical Agro-Bioresources, Guangxi Research Center for Microbial and Enzyme Engineering Technology, College of Life Science and Technology, Guangxi University, Nanning 530005, China

**Keywords:** *Methylorubrum rhodesianum* MB200, comparative genomics, carbon metabolism, methanol

## Abstract

Methylotrophic bacteria are widely distributed in nature and can be applied in bioconversion because of their ability to use one-carbon source. The aim of this study was to investigate the mechanism underlying utilization of high methanol content and other carbon sources by *Methylorubrum rhodesianum* strain MB200 via comparative genomics and analysis of carbon metabolism pathway. The genomic analysis revealed that the strain MB200 had a genome size of 5.7 Mb and two plasmids. Its genome was presented and compared with that of the 25 fully sequenced strains of *Methylobacterium* genus. Comparative genomics revealed that the *Methylorubrum* strains had closer collinearity, more shared orthogroups, and more conservative MDH cluster. The transcriptome analysis of the strain MB200 in the presence of various carbon sources revealed that a battery of genes was involved in the methanol metabolism. These genes are involved in the following functions: carbon fixation, electron transfer chain, ATP energy release, and resistance to oxidation. Particularly, the central carbon metabolism pathway of the strain MB200 was reconstructed to reflect the possible reality of the carbon metabolism, including ethanol metabolism. Partial propionate metabolism involved in ethyl malonyl-CoA (EMC) pathway might help to relieve the restriction of the serine cycle. In addition, the glycine cleavage system (GCS) was observed to participate in the central carbon metabolism pathway. The study revealed the coordination of several metabolic pathways, where various carbon sources could induce associated metabolic pathways. To the best of our knowledge, this is the first study providing a more comprehensive understanding of the central carbon metabolism in *Methylorubrum.* This study provided a reference for potential synthetic and industrial applications of this genus and its use as chassis cells.

## 1. Introduction

*Methylobacterium* was first reported in 1976 by Patt et al. This genus included a majority of pink-pigmented facultative methylotrophic bacteria. Some of the *Methylobacterium* strains can utilize methylated compounds such as methanol, methylated amines, methane halides, and methylated sulfur substances [1]. The genus is ubiquitous in nature and commonly found in freshwater, terrestrial, and plant-associated habitats, mine tailings, and marine waters [2,3,4,5,6,7,8]. Over the last century, hundreds of *Methylobacterium* strains have been identified by studying their morphology, physiology, and molecular biology. At present, there are 62 published valid species of *Methylobacterium*; they are divided into A, B, and C clades. Among them, the clade A contains 36 species and is considered the core group of *Methylobacterium*. The clade B was redefined as *Methylorubrum*, which included 10 species with the type strain *M. extorquens* AM1. Almost all *Methylorubrum* strains can use methanol or methylamine as the only carbon source. However, clade B is not reported to nodulate legumes. The clade C has relatively higher %(G+C) content and larger genome size than other clades. There are many subclades under each clade, reflecting the genomic diversity of strains within the genus [9]. As early as 1998, Ludmila et al. proposed that the genes involved in C1 transfer enzymes and coenzymes unique for their group, particularly the genes involved in the primary oxidation module of methanol, are the common boundary genes for methylotrophic bacteria and methanogenic archaea. Important pathways such as tetrahydrofolate [H_4_F/THF] and tetrahydromethanopterin/methofuran [H_4_MPT/M(Y)FR]-mediated C1 transfer pathways can coexist, which was clearly demonstrated in strain *M. extorquens* AM1 [10,11]. These pathways coexist with folate biosynthesis and methane metabolism. Simultaneously, *M. extorquens* AM1 contains tricarboxylic acid cycle (TCA), serine cycle, ethyl malonyl-CoA (EMC pathway, known to be present in 17% of prokaryotes), and various metabolic pathways of amino acids including alanine, lysine, and valine. *Methylobacterium* can metabolize single-carbon substrates (methanol, methylamine, etc.) similar to methanogenic archaea and multicarbon compounds (ethanol, glycerol, succinic acid, xylose, etc.) similar to many other non-methylotrophic bacteria [12,13]. *Methylobacterium* strains can produce many compounds including poly-beta-hydroxybutyrate (PHB), serine, uncommon dicarboxylic acid syntheses, proteins such as mevalonate, methoxybenzoic acid, 2-hydroxyisobutyric acid, and 1-butanol. The concentration and output of these compounds have been competitive [14].

*M. rhodesianum* MB200 was isolated in 2007 from a household biodigester. It was cultured in minimal medium with added methanol or succinate. It was observed that the strain MB200 can utilize and tolerate a high concentration of methanol and succinate (15–30 g/L). Moreover, the strain MB200 can use ethanol and a small amount of formaldehyde as carbon sources. Previous studies reported that the strain MB200 overexpressing serine hydroxymethyl transferase (*glyA*) and methanol dehydrogenase (*mxaF*) could increase the yield of the L-serine [15,16]. The deletion of 6-phosphogluconate dehydrogenase gene (*gnd*) effectively promotes L-serine biosynthesis [17]. These studies also indicate that pCM80 and pk18mob plasmid could stably inherit in strain MB200. As a member of *Methylorubrum*, the genome of the strain MB200 shared 62.94% collinearity with that of *M. extorquens* AM1. Meanwhile, the strain MB200 exhibited excellent growth traits after utilizing and tolerating high concentrations of methanol or succinate. It was also found that other strains of the *M. rhodesianum* genu could metabolize high concentration methanol, but the metabolic mechanism remains unclear [18]. This adaptive growth ability led us to further study the relationship between carbon metabolism and carbon flux in this genus. However, to date, the genome of the strain MB200 has not been studied, and knowledge about its physiology, biochemistry, and bacterial classification status is scarce.

The aim of this study was to explore the mechanism and characteristics of carbon metabolism in the strain MB200. In this study, we presented the complete genomic map of the strain MB200 and extensively compared its genome with that of 25 other *Methylobacterium* strains. We determined the core orthogroups shared by these 26 strains, analyzed the phylogenetic affiliations of methanol dehydrogenase (MDH) cluster genes and alcohol dehydrogenase (ADH) genes, and studied the genome collinearity between *Methylorubrum* and non-*Methylorubrum* strains. Furthermore, we studied the transcriptome of the strain MB200 grown under various carbon sources, explored the unique genes in the presence of methanol, and identified the common up- and down-regulated genes using succinate as the control carbon source. Succinate is the intermediate metabolite of methanol metabolism and ensures good growth of the strain MB200. Finally, based on all the available information, the central carbon metabolism pathway of the strain MB200 was reconstructed with more than 140 genes, and their response values under various carbon sources were displayed. To the best of our knowledge, this is the first study to report that ethanol degradation metabolism and some propionate metabolism pathways included in EMC play a role in the carbon metabolism of the strain MB200. Moreover, the GCS was reported to participate in the central carbon metabolism pathway. This study laid a theoretical foundation for the efficient bioconversion of methanol.

## 2. Results and Discussions

### 2.1. The Growth of M. rhodesianum MB200 in the Presence of Various Carbon Sources

**Figure 1:**
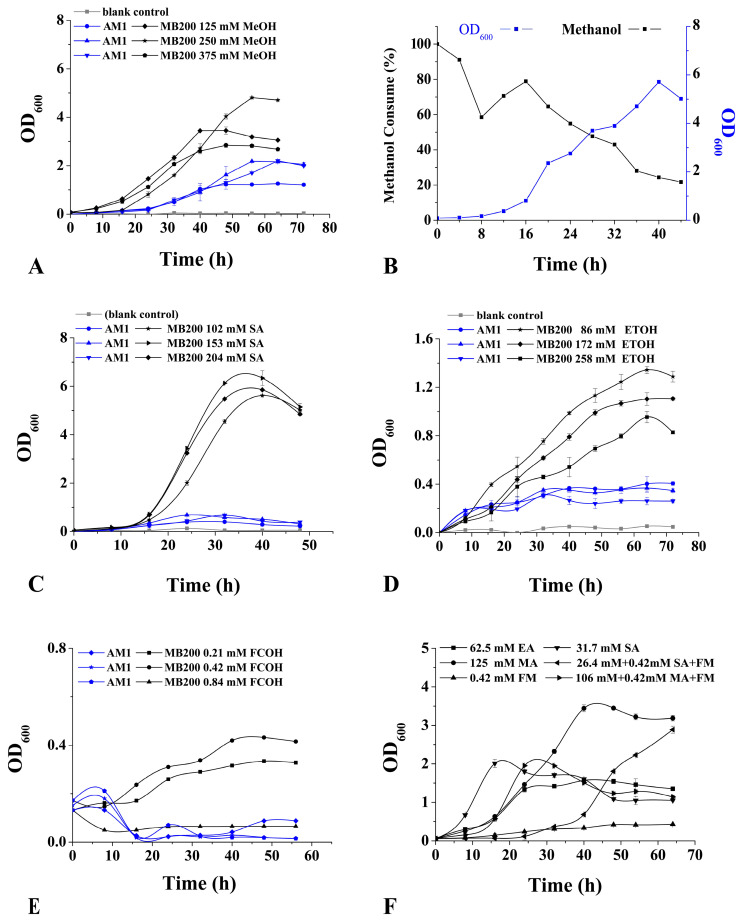
Growth profile of the strain MB200 and strain AM1. (**A**) The cells were grown on methanol (125, 250, and 375 mM) as the sole carbon and energy source. (**B**) Growth curves and methanol (250 mM) consumption curves in the strain MB200. (**C**) The cells were grown on succinate (102, 153, and 104 mM) as the sole carbon and energy source. (**D**) The cells were grown on ethanol (86, 152, and 258 mM) as the sole carbon and energy source. (**E**) The cells were grown on formaldehyde (0.21, 0.42, and 0.84 mM) as the sole carbon and energy source. (**F**) Growth profiles of the strain MB200 cultured for RNA sequencing in the presence of six carbon sources. Data are represented as mean ± standard deviation calculated from three biological replicates.

Under the supplementation of 250 mM (1% *v*/*v*) methanol, the maximum OD_600_ of the strain MB200 was 4.8, and the maximum growth rate was 0.168 per h. The OD_600_ still remained at 2.8 when the methanol concentration was 375 mM. The strain MB200 grew relatively well in the minimal medium for *Methylobacterium*, and its growth rate was higher than that of most synthetic methylotrophic strains (mainly *Escherichia coli*), except the strains that need other substrates to grow in the presence of methanol [19,20,21] (Figure 1A). The strain MB200 consumed nearly 40% of 250 mM methanol in the first 8 h and then released a little methanol into the supernatant. As the cell growth increased, methanol content decreased. At last, 80% methanol was consumed when the stabilization period reached (Figure 1B). With succinate as the carbon source, the strain MB200 could grow in the presence of 102–204 mM succinate with the highest OD_600_ being close to 7.0. However, the strain AM1 exhibited poor growth at these three concentrations of succinate (102, 153, and 204 mM) (Figure 1C). Interestingly, the strain MB200 could grow well on ethanol (258 mM) and weakly utilized formaldehyde at low concentration (0.42 mM) (Figure 1D,E).

### 2.2. Whole-Genomic Analysis of the Strain MB200

**Figure 2:**
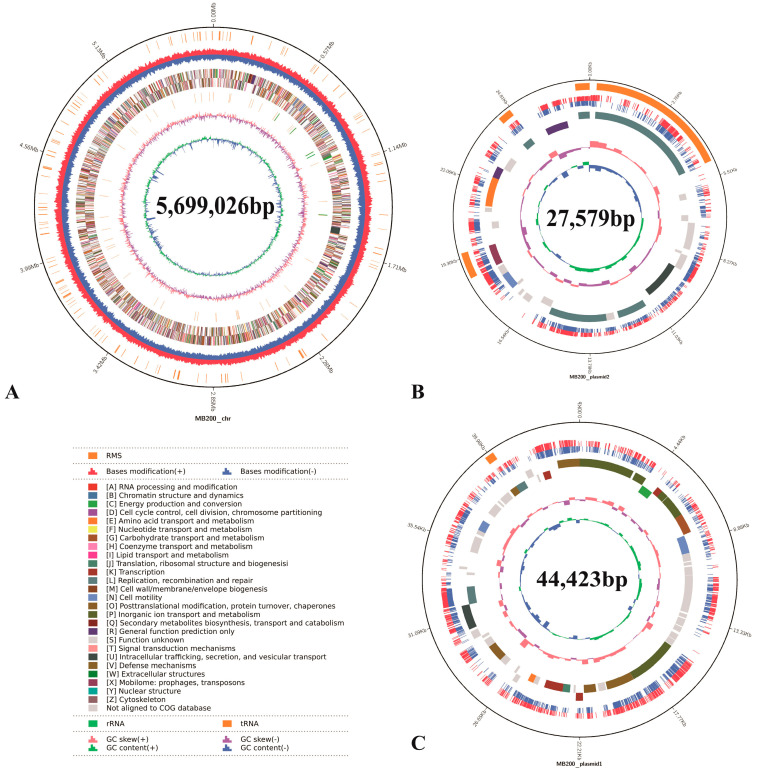
Graphical map of the genome (**A**) and two plasmids (**B**,**C**) of the strain MB200. Successive circles from the center to outside: 1, GC content; 2, GC skew (positive in pink and negative in purple); 3, rRNA in green bars and tRNA in yellow bars; 4, all genes colored by functional categories according COG classification, listed in the lower left; 5, modification of bases (positive in red and negative in blue); 6, restrictive modification of system-related enzymes; and 7, scale marks of genomes. Plasmids are not shown to scale.

The genome of the strain MB200 contains one chromosome containing 5,699,026 base pairs (bp) and G+C content of 69.46% and two plasmids of sizes 44,423 and 27,579 bp, respectively. The general genomic features of the strain MB200 were annotated in 5761 CDS, 61 tRNA, and 15 rRNA (5 each of 5S, 16S, and 23S rRNAs) genes (Figure 2). Plasmid 1 (Figure 2B) contained arsenic-detoxification-related genes and a site-specific DNA-methyltransferase and glutathione-dependent formyl dehydrogenase associated with methyltion function. Overall, 23 categories were assigned as per COGs; the top three of the items were general function prediction, signal transduction mechanisms, and cell wall/membrane/envelope biology. The predicted CRISPR sequences contained 19 GIs with a total length of 438,669 bp. GI15 contains genes related to methylotrophy. Overall, five prophage sequence regions from the strain MB200 were identified. Genes of these regions are involved in metabolic functions, transcription factors, and stress proteins. These regions may help to enhance adaptability and adhesion of the strain MB200 to surrounding environment (Supplementary Table S2). The capacity to grow in the presence of dichloromethane (DCM) has been demonstrated in *M. rhodesianum* H13 [22]. However, the strain MB200 cannot grow on DCM, and the *dcm* gene cluster was not present in the genome of the strain MB200. It was believed that the *dcm* gene cluster spread via horizontal gene transfer events.

### 2.3. Synteny Analysis

**Figure 3:**
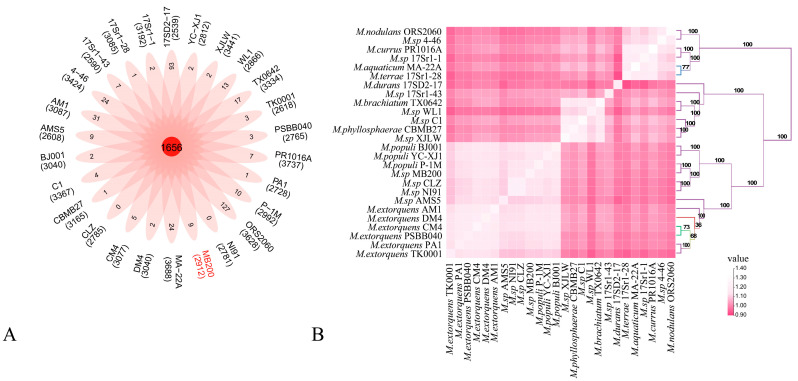
Comparative genomic study of 26 *Methylobacterium* strains with complete sequence. (**A**) Analysis of orthogroups of the 26 *Methylobacterium* strains. The 1656 core orthogroups shared by all strains are indicated in the center (red), and the unique orthogroups are indicated on each petal. (**B**) Correlation analysis of the orthogroups among 26 strains. The more the occurrence of same orthogroups, the higher the *p*-value. Phylogenetic tree on the right shows the evolutionary relationship among the 26 *Methylobacterium* strains.

To assess the collinear relationship between *Methylrubrum* (clade B) and non-*Methylrubrum* members, 12 strains were selected and divided into 2 groups. The strains used for synteny analysis are given in Table 1. The genomes of seven non-*methylrubrum* strains were compared in pairs. In Figure S6, weak collinear blocks alongside thin connecting lines between two strains indicated that the evolutionary relationship between these species was distant. Among them, *M. brachiatum* TX0642 and *M. phyllosphaerar* CBMB27 (clade A4), as well as *M. terrae* 17Sr1-1 and *M. currus* PR1016A (clade C1) exhibited the highest collinearity of 45.8% and 49.8%, respectively. All of them had a rather long evolutionary distance from the strain MB200. The genomes of six *Methylrubrum* strains were compared in pairs. Good collinearity was displayed in the *Methylrubrum* group, and the collinear blocks were obvious. The collinearity between the two strains could reach an average percentage of >70%, which indicated that their genomic correlation and the degree of gene conservation might be higher. However, the genome of *M. extorquens* TK0001 exhibited an opposite collinearity with other strains, possibly because of a genome inversion event. *Methylorubrum* strains were clustered into one branch according to their 16S rRNA (Figure 3B), connected with the pink–white square in the lower right corner on the heatmap. It was implied that they have shared more orthogroups and had closer phylogenetic affiliation.

### 2.4. Orthogroup Analysis

According to the results exported by OrthoFinder, there were 146,275 genes in total among the 26 strains (Table 1). Approximately 95.7% of them participated in orthologous protein clustering, generating 10,925 orthogroups. Overall, 1656 orthogroups constituted the core orthogroup pool, which could also be referred to as the core OGs (a group of genes descended from a single common ancestral gene) shared by 26 strains (Figure 3A). The core OGs were collected for GO enrichment using MB200 genome as the reference. The proportion of enriched core OGs is shown in Figure S5. Among them, organic substance metabolic process (n = 408), organic cyclic compound binding (n = 293), and carbohydrate derivative binding (n = 143) are related to carbon metabolism process. Meanwhile, 9 unique OGs were observed to have an internal transcribed spacer 1, ITS1, and MobA/MobL plasmid transfer initiation proteins in the strain MB200.

### 2.5. Assessment of ADH

*Methylobacterium* can metabolize short-chain alcohols or aldehydes using various types of ADHs. The ADH family have a long history of evolution and are considered versatile in the taxon. They generally catalyze the oxidation reaction of a hydroxyl group via an electron acceptor A(ox), along with cofactors. According to the nature of the cofactor, they are divided into four types: PQQ, FAD, heme and cofactor F420, and NAD(P)^+^ [23]. *Methylobacterium* mainly contains PQQ-dependent ADH and NAD(P)^+^-dependent ADH.

MDH is the most studied and representative type I PQQ-ADH [24]. MDH oxidizes a wide range of primary alcohols such as methanol (converting it to formaldehyde). This soluble quinoprotein MDH works in a complex way and either needs PQQ or metal ions including Ga^2+^ and Mg^2+^ as the auxiliary group. A novel acidic cytochrome c (cytochrome cL) is used as the physiological electron acceptor. The Ln^3+^-dependent *xoxF* is also a MDH [25]. *Methylobacterium* could accommodate the accessory genes encoding proteins with functions required for generating an active MDH through many gene clusters. These clusters are present in various parts of the genome. The MDH cluster in the strain AM1 includes 14 genes (mxaFJGIRSACKLDEHB). This cluster is approximately 12.5 kb long [26]. The sequence similarity of these genes between the strains MB200 and AM1 ranges from 89% (*mxaC*) to 96% (*mxaF*). The MDH gene cluster of the strain MB200 has much more similarity with that of *M. populi*. *M. populi* could utilize methane [27]. MDH gene clusters of some *Methylorubrum* and some non-*Methylorubrum* strains were assessed for their collinearity. The arrangement and transcription direction of MDH gene cluster on the genome of these strains were different, particularly between *Methylorubrum* and non-*Methylorubrum* species (Figure S1C). Additionally, a skip movement for *mxaD* gene was observed in non-*Methylorubrum* species. As a hallmark of methylotrophy, the MDH gene cluster in *Methylobacterium* genome was not conservative. *XoxF* has been proven to regulate the expression of *maxF* and other genes [28]. The distribution of *xoxF* in the 26 strains was conservative; however, some strains, such as AM1, have two copies of *xoxF*. The evolutionary relationship of *xoxF* in the 26 strains is shown in Figure S1D. *xoxF1* and *xoxF2* genes were observed in the strain AM1; their sequence similarity was more than 97%. There is only one *xoxF* in the strain MB200; it exhibited approximately 50% sequence similarity with that in the strain AM1. Another PQQ-dependent alcohol dehydrogenase gene *exaA* in the strain AM1 is La^3+^- or Ga^2+^-dependent, which can independently degrade methanol when both *mxaF* and *xoxF* genes were deleted [29]. *ExaA* in the strain MB200 exhibited 96.8% sequence similarity with *exaA* in the strain AM1. The transcriptome analysis of the strain MB200 cultured under various carbon sources revealed that *xoxF* was significantly upregulated along with *xoxG* and *xoxJ* under succinate but without Ln^3+^.

Besides PQQ-dependent ADH, other NAD(P)^+^-dependent ADH is present in this genus. The conserved domain of type I PQQ-ADH attaches to the Zn^2+^-dependent “medium chain” dehydrogenase/reductase family (MDR), which is further divided into eight categories according to the types of isozymes [30]. Among the 26 strains, only 15 strains contained this protein family, and their conserved domains were distinct (Figure S2A). The strain MB200 contains three types of Zn^2+^-dependent ADH, including those encoded by *adhA* (orf00121 and orf05625) and *yahk* (orf03634). All of them catalyze the conversion of alcohols into aldehydes or ketones. Based on the conservative domain phylogenetic tree, one *yahk* gene was observed to be unique in a few strains. It was cloned from the genome of the strain MB200 and heterologously expressed via pcM80 plasmid in the strain AM1. Compared with the wild-type AM1 strain, the *yahk*-overexpressing strain exhibited excellent growth in the presence of methanol or ethanol; moreover, the caking phenomenon was improved (Figure S2).

### 2.6. Analysis of Differentially Expressed Genes

**Figure 4:**
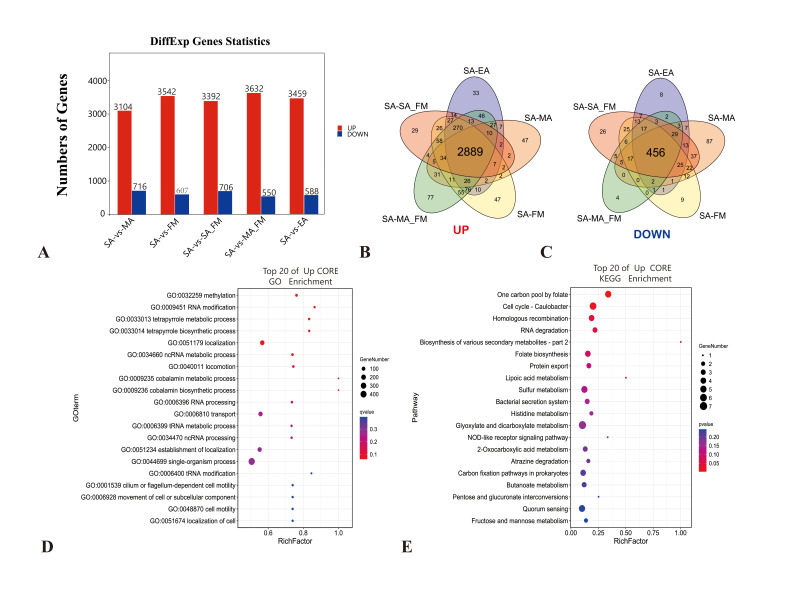
Transcriptome analysis of *M. rhodesianum* strain MB200. (**A**) The differentially expressed genes under other carbon sources relative to those under succinate (SA). For each carbon source, up- and downregulated genes are given in red and blue bars, respectively. (**B**) Venn diagram representing the core and specific enrichment of all upregulated genes under the 5 carbon sources relative to SA. (**C**) Venn diagram representing the core and specific enrichment of all downregulated genes under the 5 carbon sources relative to SA. (**D**) The degree of GO enrichment in the common upregulated genes was marked; the bubble chart shows the top 20 features. (**E**) The degree of KEGG enrichment of the core downregulated genes was marked. The bubble chart indicates the top 20 features. The closer the *p*-value is to zero, the greater the rich factor is. The higher the gene number, the more significant the enrichment.

The growth curves of the strain MB200 under six carbon sources is shown in Figure 1F. The cells were cultured for 36 h for transcriptome sequencing analysis. The differential expression analysis with succinate as the control group revealed that cells grown under methanol contained 3104 up- and 716 downregulated genes. Meanwhile, compared to succinate, other conditions including formaldehyde, ethanol, succinate and formaldehyde, methanol and formaldehyde, 3542, 3632, 3549, and 3392 genes were upregulated, and 607, 550, 588, and 706 genes were downregulated, respectively (Figure 4A). All the response genes of the six experimental groups (samples from six carbon sources) revealed that 2889 and 456 common genes were up- and downregulated, respectively (Figure 4B). 

### 2.7. GO and KEGG Enrichment Analyses of Common Response Genes

The functions of common upregulated genes were enriched in methylation (n = 160), RNA modification (n = 19), tetrapyrrole metabolic and biosynthetic process (n = 24), and localization (n = 167) (Figure 4C). Among these terms, tetrapyrrole and cobalamin are the cofactors of many formyl-related proteins. GO functions of common downregulated genes were significantly enriched in macromolecular metabolic process (n = 59) and biosynthetic process (n = 27), which indicated that the cell growth rate became worse in the presence of the other 5 carbon sources relative to succinate. KEGG enrichment analysis revealed that the common upregulated genes were mainly enriched in flagellar assembly (n = 40) and bacterial chemotaxis (n = 30), which are related to bacterial adaptation. KEGG enrichment analysis was performed on the common downregulated genes; some of the folate metabolism-related genes were clearly downregulated (n = 8) (Figure 4D). Folates are essential cofactors for one-carbon transfer reactions and are essential as the precursor for THF biosynthesis. THF participates in the reaction of transmethylation. The other enriched KEGG pathways included *Caulobacter* cell cycle (n = 6), sulfur metabolism (n = 5), and glyoxylate and dicarboxylate metabolism (n = 7).

### 2.8. Analysis of Unique Response Genes in the Presence of Methanol

The specific genes in the strain MB200 under methanol supplementation were identified. In total, 47 genes were upregulated (Figure S1A), and 87 were downregulated (Figure S1B). Except for the putative genes, the annotated unique upregulated genes included *mxaF* (5.83; log2fc relative to succinate), *fae* (3.46), *mcl* (1.68), *coxS* (1.23), *ppdK* (1.1), *metK* (1.35), *nuoF* (1.10), and SODB (1.14). Among these unique upregulated genes, *maxF* and *fae* regulate the degradation of methanol to formaldehyde; *ppdk* and *metk* could promote the production of pyruvic acid; *metk* and *nuoF* are related to energy consumption and respiration; and *coxS* is a (2Fe-2S)-binding domain protein that catalyzes the oxidation of CO to CO_2_ under aerobic conditions. The downregulated specific genes mainly included important genes involved in ATP transport and synthesis, including *virD4* (−5.14), (ATP box) ABC transporter, *glnA* (−5.11), and *atpH* (−4.63); some antioxidation-related genes such as PTGS2 (−4.46), *katG* (−5.96), and BCP (−5.8); *ldha* (−5.75) D-lactate dehydrogenase that converts lactate into pyruvate; and *soxA* (−6.68), L-cysteine S-thiosulfotransferase, involved in sulfur metabolism and converting thiosulfate into [SoxY protein]-S-(2-sulfodisulfanyl)-L-cysteine. A PTS (−5.2) 6-pyruvoyl tetrahydropterin is involved in the production of tetrahydropterin. *CdhR* (−4.52) is a cadherin. These genes are involved in the following functions: carbon fixation (*fae*, *mxaF*, and *mcl*), electron transfer chain (*metK* and *nuoF*, etc.), ATP energy release (*virD4*, ATP box, and ABC transporter), and oxidation resistance (PTGS2 and *katG*). These functional genes, along with putative proteins, play a key role in regulating the metabolism of the strain MB200 in the presence of methanol. Analyzing the unique genes in the presence of methanol is helpful to discover more genes related to the metabolism of methyl compounds.

### 2.9. Reconstruction of the Central Carbon Metabolism Pathway in the Strain MB200

**Figure 5:**
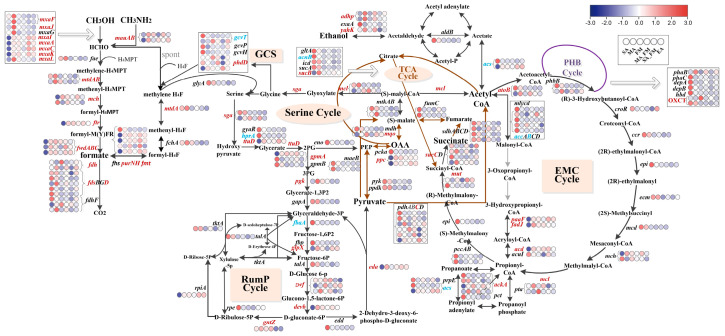
The central carbon metabolic pathways in *M. rhodesianum* strain MB200 and proposed EMC pathway containing partial propionate metabolic pathway based on our customized genomic and transcriptomic analyses. Genes encoding enzymes for each step are listed in Table S1. The common up– (red) and down– (blue) regulated genes are marked. SA: succinate; MA: methanol; FM: formaldehyde; MA_FM: methanol and formaldehyde; SA_FM: succinate and formaldehyde; EA: ethanol.

The central carbon metabolism pathway of *Methylobacterium* was under investigation for a long time to discover the key rate-limiting steps during the process when it utilizes diverse carbon sources [26,31,32]. The reconstructed central carbon metabolism pathway of the strain MB200 revealed the involvement of many cycles: primary oxidation module of methanol, serine cycle, EMC pathway including partial propionate metabolism, TCA, PHB metabolism, GCS, RuMP pathway, ethanol degradation pathway, and other modular metabolic pathways (Figure 5).

### 2.10. Heterogeneous Systems for Formaldehyde Fixation

After C1 compounds were taken up by the strain MB200, most of them were oxidized to formaldehyde by corresponding dehydrogenases such as methanol dehydrogenase in cell periplasm. Formaldehyde is an important cellular metabolite and a toxin. *Methylobacterium* strains including MB200 universally contain detoxification mechanisms, such as EfgA sensor, the primary oxidation module of methanol, and a glutathione (GSH)-dependent formaldehyde oxidation pathway.

(i) EfgA sensor.

The EfgA sensor is a conserved formaldehyde sensor encoded by *efgA* gene and arrests bacterial growth in response to high formaldehyde levels. EfgA protein has high activity and can specifically bind formaldehyde. After activation by endogenous formaldehyde, EfgA protein reduces cell growth and transcription to safeguard cells from damage through related mechanisms [33]. The *efgA* gene existed conservatively in the 26 strains. The expression of *efgA* (FPKM 471) gene in the strain MB200 was relatively high under methanol.

(ii) The primary oxidation module of methanol.

Formaldehyde is mainly fixed by the tetrahydromethanopterin (H_4_MPT) to generate methylene-H_4_MPT, and it is either a spontaneous process or can be catalyzed by a formaldehyde-activating enzyme encoded by *fae*. However, the *fae*-deletion mutant of the strain AM1 cannot grow on methanol, indicating that spontaneous condensation reaction alone is not enough to detoxify formaldehyde [34]. The methylene-H_4_MPT further gets converted into formyl-H_4_MPT by methylene-H_4_F dehydrogenase (*mtdAB*). Ultimately, formyl-H_4_MPT is converted to formate. Further, some part of formate is oxidized to CO_2_ by formate dehydrogenase as a dissimilation process and the other is transformed into formyl-H_4_F through formyltransferase (*fmt*) that takes part in the serine cycle as an assimilation process [35,36]. Recently, studies have reported that formaldehyde spontaneously combines with H_4_F; the reaction can be directly catalyzed by *mtdA*/*fch* and *ftfL* to generate formate and release energy at the same time. Some studies on the structure of the MYFR compound reported that MYFR function is the same as that of the MFR, and both are essential for H_4_MPT. MYFR is produced by various reactions and closely combines with the enzyme encoded by *ftr* to transfer formyl, thus generating formate. Its polyglutamic-acid side chain and tyramine center enable it to flexibly shuttle and transfer formyl units. The steps involved in the process are reversible [37]. Formate dehydrogenase is very important for formate to convert to carbon dioxide to complete the respiration process. An unknown NAD-dependent formate dehydrogenase (orf03626) was observed in the genome of the strain MB200, which has 65.5% sequence similarity with the formate dehydrogenase (FDH) from *Burkholderia stabilis*, as reported recently, as it favors NADP^+^ over NAD^+^ to enhance the cellular respiration [38]. In the presence of succinate, among all the genes involved in carbon metabolism, the formate dehydrogenase subunit gamma (*fdsG*) exhibited the highest FPKM value (8408.32; 12 and 35 times higher than that in the presence of methanol and ethanol, respectively) in the transcriptome. Among all six carbon sources, some of the formate dehydrogenase genes and the *glyA* gene exhibited the highest expression levels in the presence of succinate in the strain MB200.

(iii) The GSH-dependent formaldehyde oxidation pathway.

**Figure 6:**
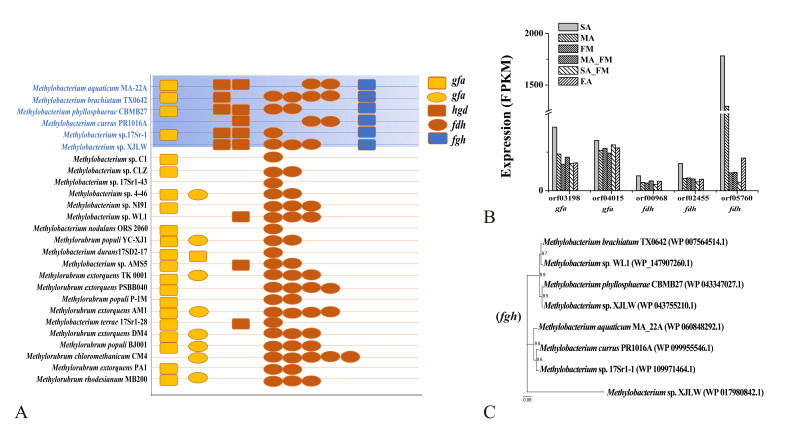
(**A**) The distribution of all genes in GSH on 26 strains and gene copies. The ordinate is the strain name: yellow square represents *gfa* gene, orange square represents *hgd* gene, orange circle represents *fdh* gene, and blue square represents *fgh* gene. The highlighted part in blue is the strain with complete GSH pathway, and the rest is the strain without *fgh* gene. (**B**) The FPKM values of genes from GSH pathway in strain MB200 grown under 6 different carbon sources. The abscissa is gene and number. Different blocks represent different carbon sources. (**C**) Phylogenetic tree of *fgh* gene in strains highlighted in blue.

GSH-dependent formaldehyde oxidation pathway usually contains three reactions and can convert formaldehyde into formate. In the strain AM1, it has been proven that the heterologous GSH-dependent formaldehyde oxidation pathway alone can make the strain survive in methanol [39]. Through protein mining, a glutathione-dependent formaldehyde dehydrogenase encoded by *fdh* (orf00968, orf02455, and orf05760) was universally found in the genome of strain MB200, as well as in that of another 25 strains, in which the conserved domain is FDH_LIKE_1 (No: CD 08283) and has 54.4–58.3% amino acid sequence consistency with the characterized *fdh* derived from *M. marinus* A45. *fdh* was proven to play the same function as *hgd* [40]. The FPKM value of *fdh* (orf05760) under succinate was 1782.93 and under methanol it was 1294.12 in the strain MB200 (Figure 6B). The genes and their copies in GSH-dependent formaldehyde oxidation pathway in 26 strains is given in Figure 6A. The highlighted blue part indicates the strains that exhibit complete GSH-dependent formaldehyde oxidation pathway, whereas the rest all lacked the *fgh* gene. *Methylobacterium* sp. XJLW is known for degrading formaldehyde. It has copies of the *fgh* gene on its genome [41]. The evolution tree of the *fgh* gene is shown in Figure 6C. GSH-dependent formaldehyde oxidation pathway might help in alleviating the toxicity of formaldehyde in this genus, and the loss of the *fgh* gene might be replaced by M(Y)FR compounds.

Collectively, these multi-mode formaldehyde detoxification mechanisms represent the key ability of the strain MB200 and *Methylorubrum* genus to degrade one-carbon compounds.

### 2.11. Serine Cycle Plays the Role of a Linkage for Other Carbon Metabolisms

Serine cycle is considered to be inefficient and requires more energy to obtain a small amount of acetyl-CoA. It seems that the carbon flux carried by this pathway is lower and is limited to some extent [13,42]. The strain MB200 exhibited rapid and better growth in the presence of methanol and exhibited faster growth when a small amount of succinate was provided; the maximum growth was reached in only 15 h (Figure 1F). There are 11 genes in a traditional serine cycle. In addition to *glyA*, there are sga-hpr-mtdA-fch and mtkA/B-ppc-mcl clusters, malate dehydrogenase (*mdh*) gene, glycerate kinase encoded by *gck*, and an enolase gene (*eno*) [26]. It was noticed that another glyoxylate reductase (*gyaR*: orf00465) gene can perform the same function as *hprA* in the strain MB200 genome. More importantly, compared with the strain AM1, *hprA* has the highest FPKM (897) among the genes of a serine pathway and has 20 extra amino acid peptides at the C-terminal. This might be related to its unique function. In the genome of the strain MB200, both *sga* and *mcl* have two copies. In the two copies of *sga*, the amino acid sequence similarity is only 48.5%; one of the orf04748 was only observed in 15 of 26 strains. Two genes of malyl-coalyase (*mcl*) are reported to have various functions in *Rhodobacter sphaeroides*; *mcl*1 is a true (3S)-malyl-CoA lyase operon and catalyzes not only the condensation of acetyl-CoA and glyoxylate, but also the cleavage of beta-methylmalyl-CoA into glyoxylate and propionyl-CoA. *mcl*2 was reported to be the (3S)-malyl-CoA thioesterase [43]. Two *mcl* genes in the strain MB200 exhibited 56.8% and 36.9% sequence similarity, respectively, with *mcl* in *R. sphaeroides*. Furthermore, the one-carbon folate-pool-related genes, particularly *gcvT* clustered by KEGG enrichment, were reported to have 4575 FPKM values under succinate (12 times more than those under methanol), which is also the primary gene of the glycine cleavage system (GCS). The GCS is a complicated loose protein interaction system that can cleave glycine into CO_2_, NH_3_, and 5,10-methylene-THF as an important C1 source [44]. The GCS was discovered to let serine cycle connect with the primary oxidation module of methanol in the strain MB200. Subsequently, two exergonic reactions (first by GCS and second by formate) occurred in the strain MB200, thus releasing more NADPH as the hydrogen donor. Schneider et al. confirmed that the glycine cleavage complex was more abundant during growth on acetate than on methanol [45]. The GCS-pathway-related genes still maintained high expression level under succinate. Further, 2PG generates 3PG through 2,3-bisphospholycerate-dependent phospholycerate mutase (GPMAB), which connects the ribulose monophosphate (RuMP) pathway. (S)-malate was imported into EMC pathway by *mcl* gene. PEP can produce pyruvate through *pyk* and *ppdk*, and pyruvate is an important intermediate metabolite and a key substance in TCA cycle, which can continuously regenerate acetyl-CoA. Except *gpmA* (orf01315) and *fumC* (orf04032), which are unique to a few strains (4 of 26 strains) including the strain MB200, most of the genes of the serine cycle are conserved among the 26 strains. In the strain MB200, the serine cycle is partially reversible and provides a platform for the transformation between single- and multi-carbon compounds, and can carry a large carbon flux and direct them toward acetyl-CoA or glycine.

### 2.12. EMC Pathway including Partial Propionate Metabolism Regulating Acetyl-CoA Redistribution

In the glyoxylate cycle, isocitrate is cleaved by isocitrate lyase to succinate and glyoxylate in one step. Due to the lack of the isocitrate lyase, *Methylobacterium* spp. enter the EMC pathway to reverse-generate succinate and glyoxylate from isocitrate. This path was verified by RémiPeyraud et al., who confirmed that two molecules of acetyl-CoA were converted into glyoxylate and succinyl-CoA along with some other metabolic intermediates [43]. Studies on *R. sphaeroides*, *M. extorquens*, and *Streptomycetes* have proven that EMC pathway can completely replace glyoxylate cycle [12]. However, Chen et al. suggested the putative degradation pathway of 3HP in EMC pathway. Acetyl-CoA produced malonyl-CoA through acetyl-CoA carboxylase (AccX). Malonyl-CoA is transformed into 3-hydroxypropionyl-CoA and 3-oxopropionyl-CoA by consuming two molecules of NADPH [14]. The enzyme function for the two steps was unclear, and the underlying pathways are unknown. Nevertheless, in the strain MB200, it was observed that 3-oxopropionyl-CoA could continue to generate acryloyl-CoA through *paaF* and further generate propanoyl-CoA to join into EMC pathway. 

Knockout of *mcd* gene in *R. sphaeroides* blocked EMC pathway; however, the strain could still be grown on a medium containing only succinate [46]. Peyraud et al., revealed that the intermediate products of EMC pathway slightly changed when the strain AM1 was cultured under succinate and methanol in turn [47]. Given these findings, this putative degradation pathway of 3HP in EMC pathway probably helps in transferring acetyl-CoA to make serine cycle unrestricted, as EMC pathway would involve many reaction steps and may have low metabolic speed.

The production of malonyl-CoA was catalyzed by several genes, which exhibited a relatively high FPKM value under succinate, particularly for *mlycd*, for which the FPKM value reached 1261. Genes of this pathway existed conservatively among 26 strains, and only *acuI* was clustered in 14 strains. *PaaF*, *fadG*, *ackA*, *accB*, and *acs* were the common up- and downregulated genes, which provided evidence for the existence of this pathway. In addition, malonyl-CoA is an important precursor of fatty acid metabolism to form cellular lipids, which corresponds to higher growth under succinate.

Generally, PHB pathway exhibited co-occurrence with EMC pathway in *Methylobacterium*. It has been revealed that PHB is synthesized from the condensation of two acetoacetyl-CoA molecules, which is catalyzed by *phaB*, and can serves as a carbon source reserve for nutritional deficiency [38]. It was observed that the FPKM of *phaB* (acetoacetyl-CoA reductase) reached 2043 under methanol and 558 under ethanol, which was 8.6- and 2.3-times more than that under succinate, respectively. This indicated that more PHB would be accumulated under methanol and ethanol.

### 2.13. TCA Cycle

In TCA cycle, pyruvate is oxidized and converted into acetyl-CoA through citrate (C3), and energy is released. According to the reconstructed metabolic pathway of the strain MB200, succinate would be degraded to fumarate and then to (S)-malate; serine cycle would be interrupted and flown into two directions via (S)-malate, generating OAA in one direction and acetyl-CoA in the other direction. The two substrates would then generate citrate. The genes involved in TCA cycle exhibited high transcript levels under succinate, and the FPKM value of citratesynthase (*gltA*) reached 475.91, which was nearly four times than that under methanol. As OAA is the intermediate product and is decomposed, TCA cycle in methanol metabolism seems to be inactive compared with that in succinate metabolism.

### 2.14. The Degradation Pathway of Ethanol

The degradation pathway of ethanol was included in the central carbon metabolism. When ethanol enters the cell, alcohol dehydrogenase catalyzes the conversion of ethanol to acetaldehyde. Acetaldehyde is further converted to acetate; the process is catalyzed by acetaldehyde dehydrogenase encoded by *aldB*, which is NADP-dependent. Finally, acetate is converted to acetyl-CoA; the reaction is catalyzed by acetyl-CoA synthetase encoded by *acsA*. *AcsA* has two copies in the strain MB200; the gene numbers are orf02677 and orf04983. When orf04983 was knocked out, the logarithmic period was shortened by approximately one-fold, and the growth in 0.5% (*v*/*v*) ethanol was increased by 12% (Figure S3). The growth of orf02677-knockout mutant and wild type was similar. The knockout experiments of two genes separately revealed that no significant difference existed in the growth under succinate and methanol, indicating that *acsA* might not participate in the regulation of central carbon metabolism. The failure to increase the final growth may be related with the regulation of genes under ethanol. In conclusion, the regulation of acetyl-CoA in the strain MB200 is agile and can be redistributed by surrounding carbon metabolisms. It is worth noting that acetyl-CoA may have four flow directions in the central carbon metabolic map. Each direction usually has more than two regulatory genes, and most of them exhibit opposite expression levels on the transcriptome.

### 2.15. Ribulose Monophosphate (RuMP) Pathway with Functional Loss

As the way of regeneration of 5-carbon sugar, RuMP is the continuation of the glycolysis pathway. It is closely related with bacterial photosynthesis, which can provide NADPH and ATP and maintain the stability of GSH. The intermediate products are the substrates of many other pathway reactions. According to the matched genes on RuMP cycle, the strain MB200 and most *Methylobacterium* strains lack the key hexulose-6-phosphate synthase (HPS) and hexulose-6-phosphate isomerase (PHI), which are unique to obligate methylotrophic bacteria [48]. PHI catalyzed the reaction between D-ribulose5-phosphate and formaldehyde to produce D-arabino-hex-3-ulose6-phosphate in one step, which would be converted to fructose-6-phosphate by HPS to shorten the pathway processes. However, compared with the obligate methylotrophs, D-ribulose5-phosphate would generate D-xylulose-5-phosphatec in the strain MB200 (Figure 5). Transcripts of genes in RuMP pathway still maintained the highest level under succinate. Among the genes related to RuMP pathway, the FPKM of *fbaA* reached 2636.76 under succinate, which was 16 times more than that under other carbon sources. The RuMP pathway in the strain MB200 tends to produce complex polysaccharides such as sedoheptulose-7-phosphate to immobilize biomass, providing precursor materials for the generation of some more complex compounds. According to a previous study, *devB* is negatively regulated by succinate, which is not conducive to the circulation as it forms toxic intermediate metabolites such as D-glucono-1,5-lactone-6P [49]. Through incorporating the heterologous RuMP pathway in the strain AM1, the cell growth rate of the mutant strain increased by 16.5%; its OD_600_ was less than that of the strain MB200 under methanol. This may indicate that the RuMP cycle is related to other pathways [48].

According to the reconstructed graph (Figure 5), the possible main carbon flow direction of the strain MB200 under various carbon sources was revealed. In the presence of methanol and formaldehyde, the primary oxidation module of methanol is followed; some part of carbon flows toward formate to generate carbon dioxide, and another part flows toward serine cycle along with TCA cycle and EMC pathway and tends to form more PHB. In the presence of succinate, ethanol, or acetate, TCA cycle or EMC pathway is followed as assimilation. Some part of carbon flows toward the primary oxidation module of methanol through serine cycle and GCS to generate carbon dioxide; less PHB is produced and tends to decompose for increasing growth.

## 3. Materials and Methods

### 3.1. Bacterial Strains and Growth Conditions

The strains *M. rhodesianum* MB200 and *M. extorquen* AM1 were cultured at 30 °C in modified version of ATCC medium, i.e., 396 methylamine-salts medium (MM). MM contains three parts: the phosphate salts solution [(NH_4_) _2_HPO_4_ 3 g/L, K_2_HPO_4_ 2 g/L, and NaCl 1 g/L in 1000 mL distilled water; pH 7.0), solution A (MgSO_4_·7H_2_O 20 g/L, CaCl_2_·2H_2_O 2 g/L, and FeSO_4_ 2 g/L in 100 mL distilled water), and solution B (MnSO_4_·7H_2_O 0.5 g/L, Na_2_MoO_4_·2H_2_O 0.5 g/L, and ZnSO_4_·7H_2_O 0.5 g/L in 100 mL distilled water). Trace biotin was added at a ratio of 1/10,000 in the medium [50]. The following concentrations of each carbon source were added to MM medium before use: formaldehyde (0.21, 0.42, and 0.84 mM; “FM”), methanol (125, 250, and 375 mM; “MA”), ethanol (86, 172, and 258 mM; “EA”), and succinate (102,153, and 204 mM; “SA”). *E. coli* strains were grown on Luria–Bertani (LB) medium. The antibiotics were added at the following final concentrations: ampicillin 50 μg/mL, kanamycin 50 μg/mL, and tetracycline 30 μg/mL.

### 3.2. Growth Experiments

For each growth experiment, the cells were cultured till mid-log phase. For further growth curve experiment, 100 mL culture suspension with OD_600_ of 0.1 was added to a 250 mL Erlenmeyer flask. The OD_600_ was measured every 8 h using a spectrophotometer. The experiment was performed in triplicates. For genome sequencing, the cells were cultured as follows. The cells of strain MB200 grown under methanol were collected in mid-log phase. Microscopic examination, 16S rRNA amplification, and sequencing were performed to assess any contamination. The bacterial suspension (1 L) was centrifuged at 6000 rpm (Hermle Z36HK, Stuttgart, Germany) for 8 min and washed with PBS (pH 7.2–7.4) 3–4 times until the supernatant was clear. The supernatant was discarded, and the cells were quickly frozen in liquid nitrogen and stored at −80 °C till use. For RNA sequencing, the cells were cultured as follows. Considering 125 mM methanol as the reference for the normal growth of the strain MB200, the carbon and nitrogen ratio was controlled to 2.35:1 so that the same carbon content was maintained. The cells were collected after 36 h of incubation. The control group was treated with succinate (SA) (31.7 mM). The treatment groups were treated with methanol (MA) (125 mM), formaldehyde (FM) (0.42 mM), ethanol (EA) (62.5 mM), methanol and formaldehyde (MA_FM) (106 mM + 0.42 mM, respectively), and succinate and formaldehyde (SA_FM) (26.4 mM + 0.42 mM, respectively).

#### GC-MS Analysis

The strain MB200 was grown in 250 mL MM liquid medium supplemented with 1% methanol as the carbon source and incubated at 30 °C for 48 h. Overall, 1 mL of the culture medium was collected every 8 h and immediately centrifuged (8000× *g*; 2 min). The supernatant was filtered through a Sartorius Minisart filter (pore size 0.2 mm) before analysis. SHIMADZU GC2010 GC-MS was fitted with a capillary column SH-RTX-Wax (30 m × 250 μm i.d. and 0.25 μm film thickness). The conditions were injector temperature: 250 °C, transfer line temperature: 250 °C, and injection volume: 0.4 μL. The experiment was operated in a split module, and the splitting ratio was 40:1. The GC was programmed as follows: 5 min at 45 °C, increase to 120 °C at 16 °C/min and retention for 1 min, further increase to 220 °C at 50 °C/min, and keep 1 min. The carrier gas was N_2_ (0.8 mL·min^−1^). Methanol was detected by comparing retention times with known concentration of isobutanol as internal standard for mass spectra. The residual methanol content was calculated according to the volume of fermentation liquid.

### 3.3. Whole-Genome Sequencing, Assembly, and Annotation

The genomic DNA of the strain MB200 was extracted using an E.Z.N.A. Bacterial DNA Kit (Omega, Bio-Tek, Norcross, GA, USA) according to the manufacturer’s instructions and sequenced using PacBio. For PacBio sequencing library, the insert size was longer than 10 Kb. Genomic DNA (1 μg) was sheared into 10–15 Kb fragments using a g-TUBE device from covaris. The sheared fragment size was assessed using the Fragment Analyzer device. Further, a library was constructed using the SMRTbell Express Template Preparation Kit 2.0 (Pacific Biosciences, Menlo Park, CA, USA) according to the manufacturer’s instructions. The size of the library was assessed using an Agilent 2100 Bioanalyzer System with an Agilent HS DNA Kit (Agilent Technologies, Santa Clara, CA, USA). The library was sequenced using Sequel Sequencing Kit 2.0 (PacBio), and 10-h movies were captured for each SMRT Cell 8M using the Sequel II sequencing platform [51]. PacBio subreads were processed and assembled using the Microbial Assembly (SMRTLINK8.0), HGAP4 (SMRTLINK8.0), and Canu (v1.6). pbalign (v0.4.1 default blast algorithm) software was used to compare the subreads with the assembled genome and analyze the coverage depth distribution of the genome. Approximately 3.8 Gb data were obtained, with 544,522 subreads and 7321 bp contig N50. The original reading of the strain can be obtained from Genbank through the following login number: (code after uploading). The databases NR, Swiss-Prot, COG (orthologous cluster), KEGG, GO, and Carbohydrate Active Enzyme Database (CAZy) were used for protein function annotation [52]. RNA, virulence factors, and resistance genes were determined based on the core data sets in Rfam (database reference), virulence factors of pathogenic bacteria (VFDB), and Antibiotic Resistance Gene Database (ARDB). The software trf409.legacylinux64, MinCED (v0.3.2), IslandPath-DIOMB, and PhiSpy (v2.3) were used to predict the TRF (tandem repeats), CRISPRs, gene island, and prophage of the whole genome [53].

### 3.4. Synteny Analysis and OrthoFinder

Blastp (version 2.10.1+) was used for genome sequence alignment, and Mcscanx (default parameter) was used to compare the genome of the strain MB200 with other strains in this genus. Collinear diagrams were plotted using package ggplot2. Among them, seven strains in the first group (Figure S6) were screened by 70% similarity of blast, and the six strains in the second group revealed all collinear blocks without screening. To view the shared or unique protein families of the strain MB200 and other strains in this genus, 26 completely sequenced *Methylobacterium* genomes (including the strain MB200) were clustered by running OrthoFinder, and their protein sequences were downloaded from NCBI. The accession numbers of the strains are listed in Table 1. OrthoFinder was used to cluster orthologous proteins in the genus, and each clustered protein family was given a number. The sequence similarity search tool DIAMOND BLAST was used to identify the OGs, based on single copy homologous genes of the 26 species. We extracted the single-copy gene family sequences of the 26 strains through OrthoFinder and conducted multiple sequence alignments. Further, using IQtree software, we screened the best base replacement model for the processed sequence, and constructed the phylogenetic tree with the maximum likelihood method. The number of bootstrap replicates was 1000 [54]. TBtools was used to draw the diagrams [55].

### 3.5. RNA-seq Analysis

A proper amount of bacterial suspension was centrifuged; the cell pellet was washed with a proper amount of PBS (pH 7.2–7.4) many times until the bacteria were clean. PBS was removed, and the bacteria were quickly frozen in liquid nitrogen at −80 °C till use. The samples were sent to OmicShare company (Guangzhou, China) for RNA-seq. After sequencing, the FASTQ format file was obtained. The FASTQ files were subjected to quality control, and low-quality reads were removed to get “clean reads”. Bowtie2 was used to compare clean reads with the strain MB200 genome (Genbank number: PRJNA894144). HTSeq0.6.1p2 software was used for statistical analysis, and TBtools software was used to convert statistical data into FPKM values for further analysis. Data were analyzed using DESeq (version1.18.0) software. The differential analysis of RNA-seq data was performed, and the differentially expressed genes were selected by setting the multiple of gene difference more than twice (|log2FC| > 1) and *p*-value < 0.05 as the threshold values. GO and KEGG databases were used for functional annotation and enrichment analysis of differentially expressed genes [56].

### 3.6. Heterologous Expression in the Strain the Strain AM1 and Gene Knock-In in the Strain MB200

Overall, 10 mL of bacterial suspension was taken. The genome of the strain MB200 was extracted using the E.Z.N.A. Bacterial DNA Kit (Omega, Bio-Tek, Norcross, GA, USA) as per the manufacturer’s instructions. *Yahk* gene (GeneNo.: orf03634) was amplified using the primers given in Table 2. PCM80 (GenBank: AF327716.1) plasmid was extracted from *E. coli* DH5α cells using the SanPrep column plasmid Mini-Preps Kit. The plasmid and gene were digested using *PstI* and *EcoRI* fast-cutting enzyme (Takara), ligated by T4DNA ligase at 16 °C for 12 h, and used to transform *E. coli* DH5α competent cells by heat shock method. The cells were plated on LB plate containing Tc, and the verified clones were selected. The extracted plasmid was transferred into the strain AM1 using an electroporation device [57], and the cells were plated on MM medium containing Tc (with 4 g/L methanol) to produce hey-AM1 (heterologous overexpression of *yahk*) cells. *acsA1* (GeneNo: orf02677) and *acsA2* (GeneNo: orf 4983) were cloned from the strain MB200 genome using the primers given in Table 2, and the intermediate fragments of the two genes (accounting for half of the total length) were amplified from DH5α. The SanPrep column plasmid Mini-Preps Kit was used to extract pk18mob plasmid, which carried a kanamycin resistance gene. The gene and plasmid were digested using *EcoRI* and *XbaI* and ligated using T4DNA ligase at 16 °C for 12 h. The plasmid was used to transform DH5αΧ competent cells by heat shock method, and the cells were plated on LB plate containing kanamycin. The correct clones were picked and verified. The plasmid was extracted and transferred into the strain MB200. Homologous recombination and single exchange were performed, and the cells were plated on MM medium containing kanamycin (with 4 g/L ethanol) to obtain mutants ΔacsA1 and ΔacsA2. The comparative growth experiment was completed in the Erlenmeyer flask according to the method described above.

### 3.7. Detection of the Strain MB200 in Sudan Black B Stain

Overall, 1 mL bacterial suspension in logarithmic growth period was centrifuged at 8000 rpm for 2 min. The cell pellet was washed 2–3 times with PBS, stained with 0.5% Sudan Black B for 5 min, washed with water, and decolorized with xylene for 30 s. The cells were again stained with 0.5% crocus solution for 1 min, washed, dried, and examined using a microscope. The PHB granules in the cell appeared blue.

## 4. Conclusions

In this study, the mechanisms underlying the utilization of a high concentration of methanol and other carbon sources by strain MB200 were assessed. Comparative genomic analysis revealed the genomic diversity of *Methylobacterium* genus. The genomic collinearity within *Methylorubrum* spp. was high, and the MDH gene cluster was relatively conservative. *Methylorubrum* strains have more common orthogroups. The strain MB200 contains more than 140 genes related to carbon metabolism such as *maxF*, *fae*, *yahk*, *fdh* (orf03626), and *hprA*, which might promote its carbon fixation capacity and flexible regulatory ability of acetyl-CoA. The reconstructed central carbon metabolic pathway revealed that each metabolic module has its own characteristics and roles. As succinate is the intermediate metabolite of methanol metabolism and ensures good growth of the strain MB200. Therefore, the strain MB200 might induce partial succinate-mediated growth mode to maintain its good growth state when using methanol as the sole carbon source. Moreover, to the best of our knowledge, this is the first study reporting that a part of the propionate pathway in EMC pathway appears to help in transferring acetyl-CoA. The ethanol degradation pathway exhibits less influence on the metabolism of methanol and succinate. Our study provided a more comprehensive understanding and theoretical basis for carbon metabolism in methylotrophic bacteria and verified one-carbon utilization by natural methylotrophic bacteria.

## Figures and Tables

**Table 1:** The details of samples used for comparative genomic analysis.

Genome Name/Sample Name	NCBI Assembly Accession	Gene Count	Genome Size (Mb)
*Methylorubrum rhodesianum* MB200 ^S^	In this study (PRJNA894144)	5771	5.77
*Methylobacterium* sp. NI91	GCA_011212365.1	5150	5.41
*Methylobacterium phyllosphaerae* CBMB ^S^	GCA_001936175.1	5886	6.32
*Methylobacterium* sp. 4–46	GCA_000019365.1	7125	7.66
*Methylobacterium currus* PR1016A ^S^	GCA_003058325.1	7460	6.55
*Methylobacterium* sp. XJLW	GCA_003254375.1	6457	6.67
*Methylobacterium* sp. C1	GCA_001854385.1	6177	6.46
*Methylobacterium* sp. CLZ ^S^	GCA_011212505.1	5147	5.41
*Methylobacterium* sp. 17Sr1-43	GCA_003173735.1	5273	5.54
*Methylobacterium brachiatum* TX0642 ^S^	GCA_003697185.1	6359	6.34
*Methylobacterium aquaticum* MA-22A	GCA_001548015.1	7148	7.56
*Methylobacterium* sp. WL1	GCA_008000895.1	6351	6.25
*Methylobacterium nodulans* ORS 2060 ^S^	GCA_000022085.1	8885	8.84
*Methylobacterium durans* 17SD2-17 ^S^	GCA_003173715.1	6788	6.79
*Methylobacterium.terrae* 17Sr1-28 ^S^	GCA_003173755.1	5757	6.16
*Methylobacterium* sp. 17Sr1-1	GCA_003173775.1	5741	6.54
*Methylobacterium* sp. AMS5	GCA_001542815.1	5183	5.44
*Methylorubrum extorquens* TK 0001 ^S^	GCA_900234795.1	5525	5.72
*Methylorubrum extorquens* PSBB040	GCA_001971665.1	5323	5.70
*Methylorubrum populi* P-1M	GCA_002355515.1	5644	5.94
*Methylorubrum extorquens* AM1 ^S^	GCA_000022685	6294	5.51
*Methylorubrum populi* YC-XJ1	GCA_006740745.1	5094	5.40
*Methylorubrum extorquens* DM4 ^S^	GCA_000083545.1	5829	5.66
*Methylorubrum populi* BJ001 ^S^	GCA_000019945.1	5848	5.80
*Methylorubrum chloromethanicum* CM4	GCA_000021845.1	6180	6.18
*Methylorubrum extorquens* PA1	GCA_000018845.1	5471	5.47

^S^ means the strain selected for synteny analysis.

**Table 2:** Primers used in this study.

Primer Names	Primer Sequence (5′–3′)
*acsA*1 F	ATTAGAATTCCCACGCCATCGTCTTCGGTG
*acsA*1 R	TAATTCTAGACGACGCGTCCGGTGATCCAG
*acsA*2 F	ATATGAATTCCGGTGGTGATCACTCACTTCG
*acsA*2 R	TATATCTAGATCGCCTCGGGCTGATTCCAGT
*yahk* F	ATCTGCAGATGTTCACCTGCACGGGCTATGC
*yahk* R	AAGAATTCTCAGGCGGCCTTGGGCAGGCTC
Km F	ATGATTGAACAAGATGGATTGCACGCAGGT
Km R	TCAGAAGAACTCGTCAAGAAGGCGATAGAA

## Data Availability

The raw data of genome and transcriptome are publicly available through the NCBI SRA database (SRA accession number: PRJNA894144).

## Supplementary Materials

The following supporting information can be downloaded at: https://www.mdpi.com/article/10.3390/ijms24087521/s1.
