# Supplementary material for: Comparative Genomic Analysis of a Methylorubrum rhodesianum MB200 Isolated from Biogas Digesters Provided New Insights into the Carbon Metabolism of Methylotrophic Bacteria
Source: Int J Mol Sci. 2023 Apr 19;24(8):7521. doi: 10.3390/ijms24087521 (PMC10138955; doi:10.3390/ijms24087521)
Supplement: Supplementary file 1 [file ijms-24-07521-s001.zip › Supplementary materials-revise.pdf]

Table S1. Carbon metabolic details of *M. rhodesianum* MB200. Genes involved in central carbon metabolism pathway and their information of strain MB200. The KEGG annotation is indicated for each gene. Transcriptomic analysis of strain MB200 under six carbon sources were presented. Results for intrastrain comparisons are complied, with FPKM set as limit.

Table S2. Gene Island and prephage region prediction of *M. rhodesianum* MB200

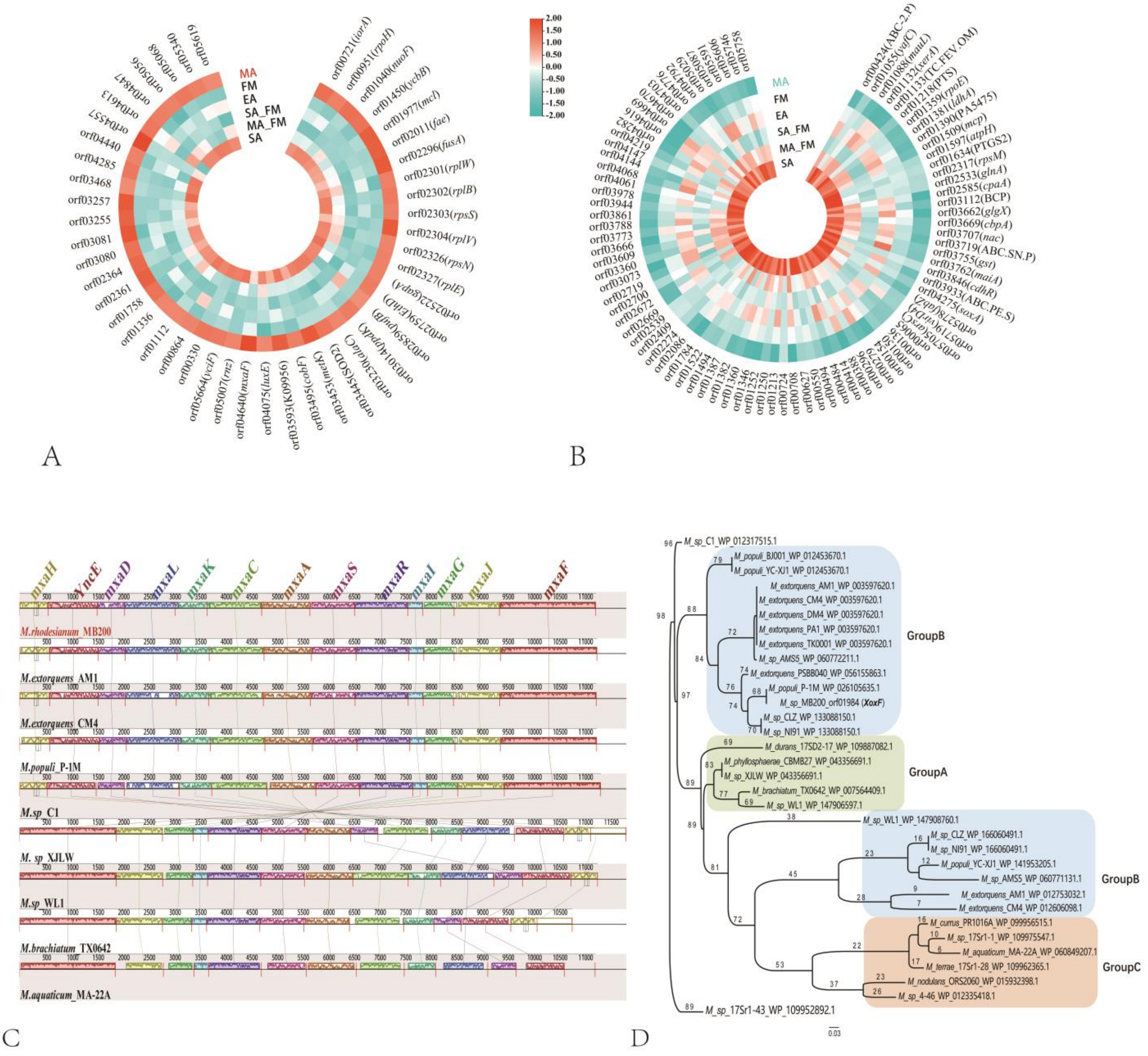

Figure S1. (A&B) Unique 47 upregulated and 87 downregulated genes under methanol

(MA) were identified based on previous transcriptome analysis. The gene number and their FPKM under 6 conditions were used to form the circular heatmap. Salmon pink represents upregulated and sky-blue represents downregulated genes. (C) Comparative methanol gene cluster analysis of partial Methylobacterium strains. The image was generated using Mauve. (D) Phylogenetic tree of *xoxF*. Multiple sequence alignment of *xoxF* family amino acids among 26 strains is given with strain names. The letters in brackets are number of amino acids of xoxf in each strain. The tree was constructed by maximum likelihood. The calculated bootstrap values are indicated at the nodes. Group B strains are highlighted in light blue, group A in light green, and group C in light yellow. Seven strains in group B have two copies of *xoxF* genes.

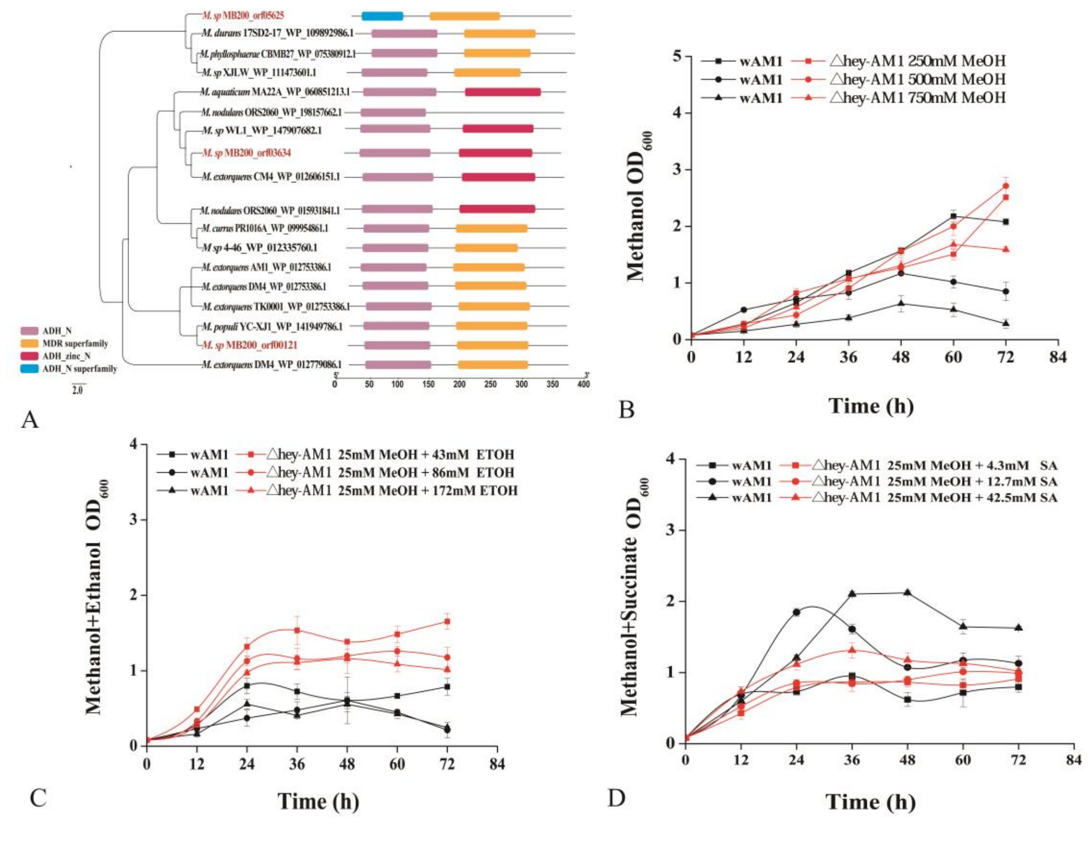

Figure S2. (A) Conserved domain and motifs in the (Zn-depentent)-ADH family cluster in 26 strains.These homologous genes were obtained from different strains. Growth of Methlorubrum extorquens AM1 (black line), hey-AM1(red line) cultured with three carbon sources. (B) The cells were grown on methonal (250, 500, and 750 mM). (C) The cells were grown on ethanol (43, 86, and 172 mM). (D) The cells were grown on succinate (4.3, 12.7, and 42.5mM).

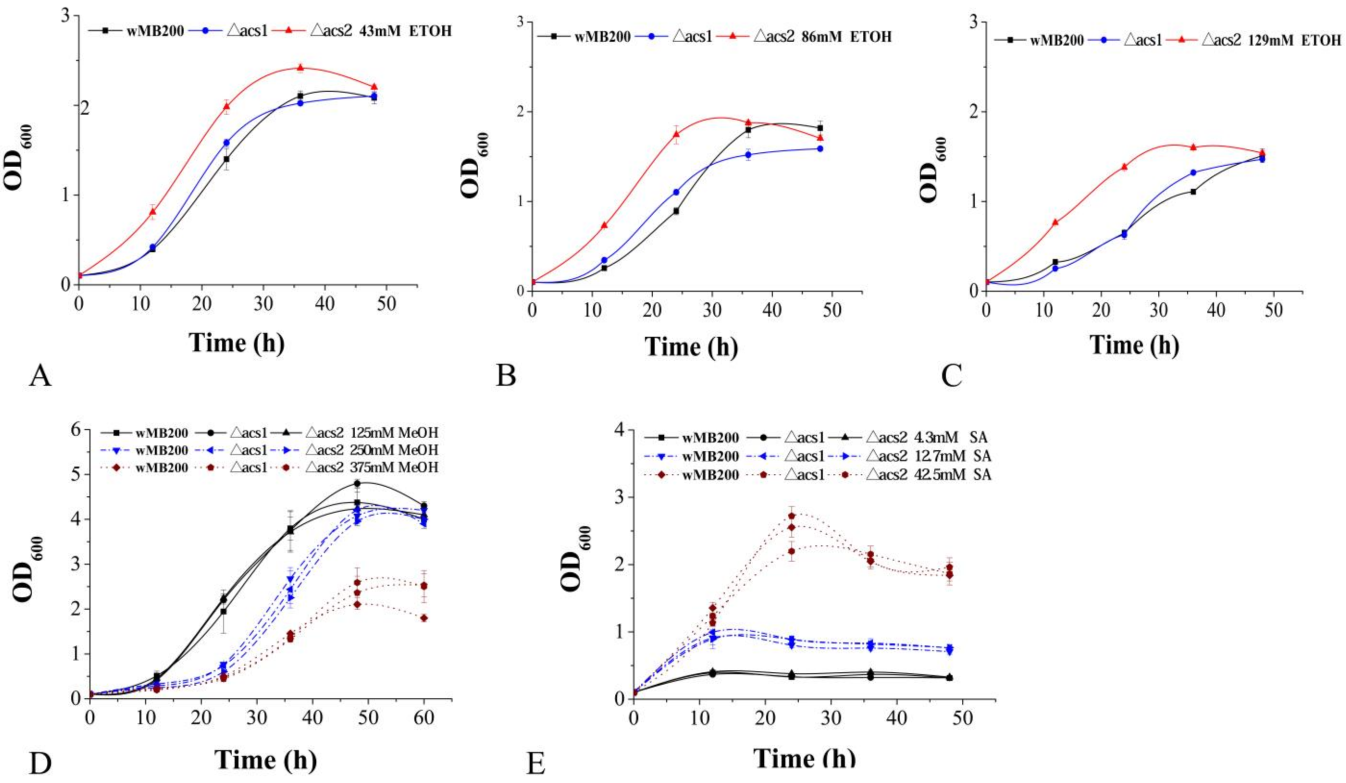

Figure S3. Growth of MB200 (black line), Δacs1 (orf02677) (blue line), and Δacs2 (orf04983) (red line) with 43 (A), 86 (B), and 129 (C) mM ethanol as the sole carbon source. (D) Growth of MB200, Δacs1, and Δacs2 with 125 (black line), 250 (blue line), and 375 (brown line) mM methanol as the sole carbon source. (E) Growth of MB200, Δacs1, and Δacs2 with 4.3 (black line), 12.7 (blue line), and 42.5 (brown line) mM succinate as the sole carbon source.

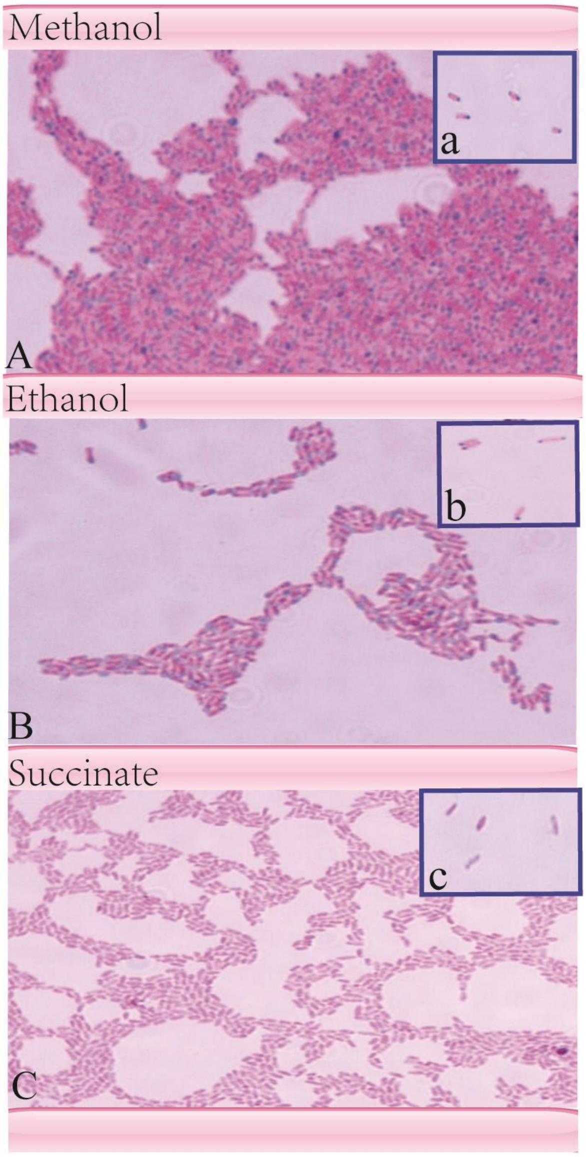

Figure S4. Microscopic examination of MB200 cells cultured with various carbon sources. The cells were stained with Sudan Black B. Figs A, B, and C show the strains cultured with methanol, ethanol, and succinate as the sole carbon sources, respectively. The squares indicated by a, b, and c show the area with single cells. The cells grown in methanol were stained blue and exhibited clear blue spots, indicating that it contained intracellular PHB granules. The cells grown in ethanol were stained blue, indicating that PHB granules were present. The cells grown in succinate were not stained in blue, indicating scarce generation of PHB granules. This is consistent with the central carbon metabolism pathway predicted in this study.

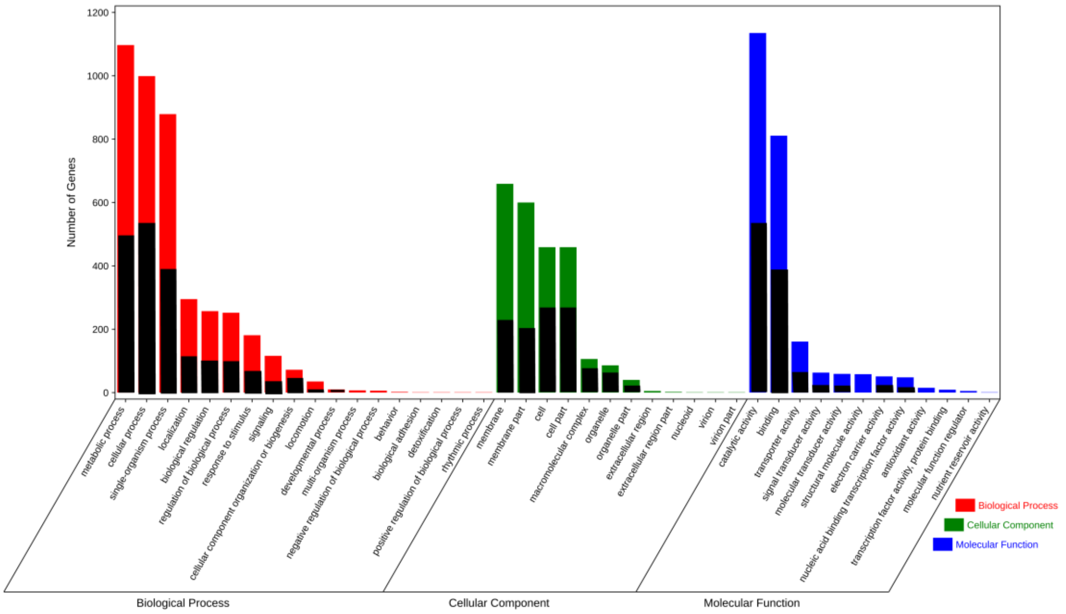

Figure S5. GO function of core pool genes of strain MB200. The black bar represents the proportion of core pool genes.

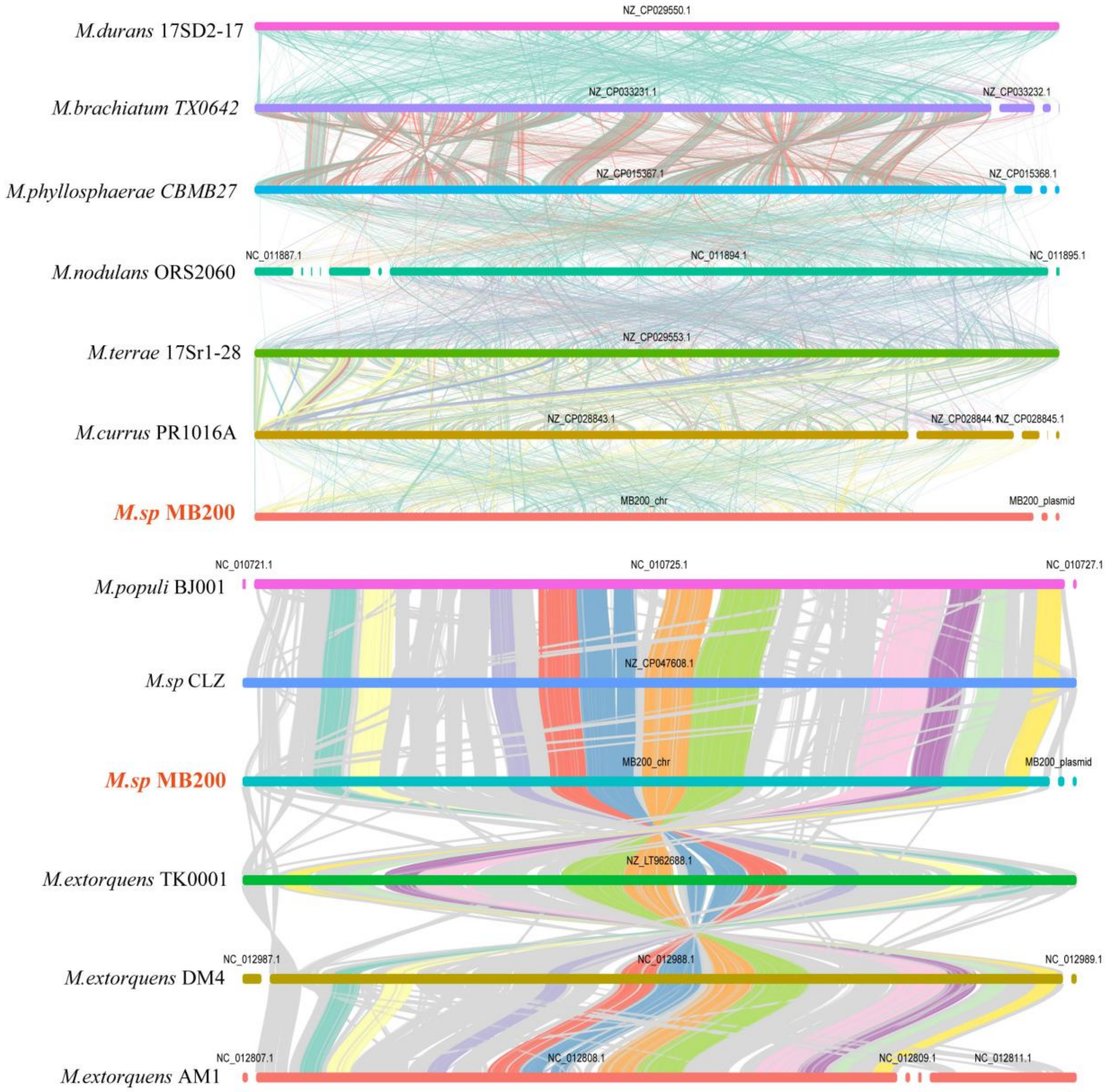

Figure S6. Synteny analysis of the genomes of 13 strains is presented in two groups; each Genbank number is displayed on the colored stick. The linkage shows a synteny line between two adjacent strains; their names match with the right. Gray lines indicate the synteny blocks. Syntenic genes are given with colored lines.
